# Person-centred care in the Dutch primary care setting: Refinement of middle-range theory by patients and professionals

**DOI:** 10.1371/journal.pone.0282802

**Published:** 2023-03-09

**Authors:** Anam Ahmed, Maria E. T. C. van den Muijsenbergh, Hubertus J. M. Vrijhoef

**Affiliations:** 1 Panaxea b.v., Amsterdam, The Netherlands; 2 Department of Primary and Community Care, Radboud University Medical Centre, Nijmegen, The Netherlands; 3 Department of Prevention and Care, Pharos: Dutch Centre of Expertise on Health Disparities, Program Prevention and Care, Utrecht, The Netherlands; 4 Department of Patient & Care, Maastricht University Medical Center, Maastricht, The Netherlands; Universitat Luzern, SWITZERLAND

## Abstract

In a previous rapid realist review (RRR) of international literature insight was provided into how, why, and under what circumstances person-centred care (PCC) in primary care works (or not) among others for people with low health literacy skills and for people with a diverse ethnic and socioeconomic background, by establishing a middle-range programme theory (PT), which describes the relationship between context items, mechanisms, and outcomes. Since the application of PCC in primary care in the Dutch setting is expected to differ from other countries, the objective of this study is to validate the items (face validity) resulting from the RRR for the Dutch setting by assessing consensus on the relevance of items. Four focus group discussions with patient representatives and patients with limited health literacy skills (n = 14), and primary care professionals (n = 11) were held partly combined with a Delphi-study. Items were added to refine the middle-range PT for the Dutch primary care setting. These items indicated that in order to optimally align care to the patient tailored supporting material that is developed together with the target group is important, next to providing tailored communication. Healthcare providers (HCPs) and patients need to have a shared vision and set up goals and action plans together. HCPs should stimulate patient’s self-efficacy, need to be aware of the patient’s (social) circumstances and work in a culturally sensitive way. Better integration between information and communications technology systems, flexible payment models, and patients access to documents, and recorded consultations should be in place. This may result in better alignment of care to the needs of patients, improved accessibility to care, improved patient’s self-efficacy, and improved health-related quality of life. On the long-term higher cost-effectiveness and a higher quality of healthcare can be realised. In conclusion, this study shows that for PCC to be effective in Dutch primary care, the PT based on international literature was refined by leaving out items and adding new items for which insufficient or sufficient consensus, respectively, was found.

## Introduction

**Fig 1:**
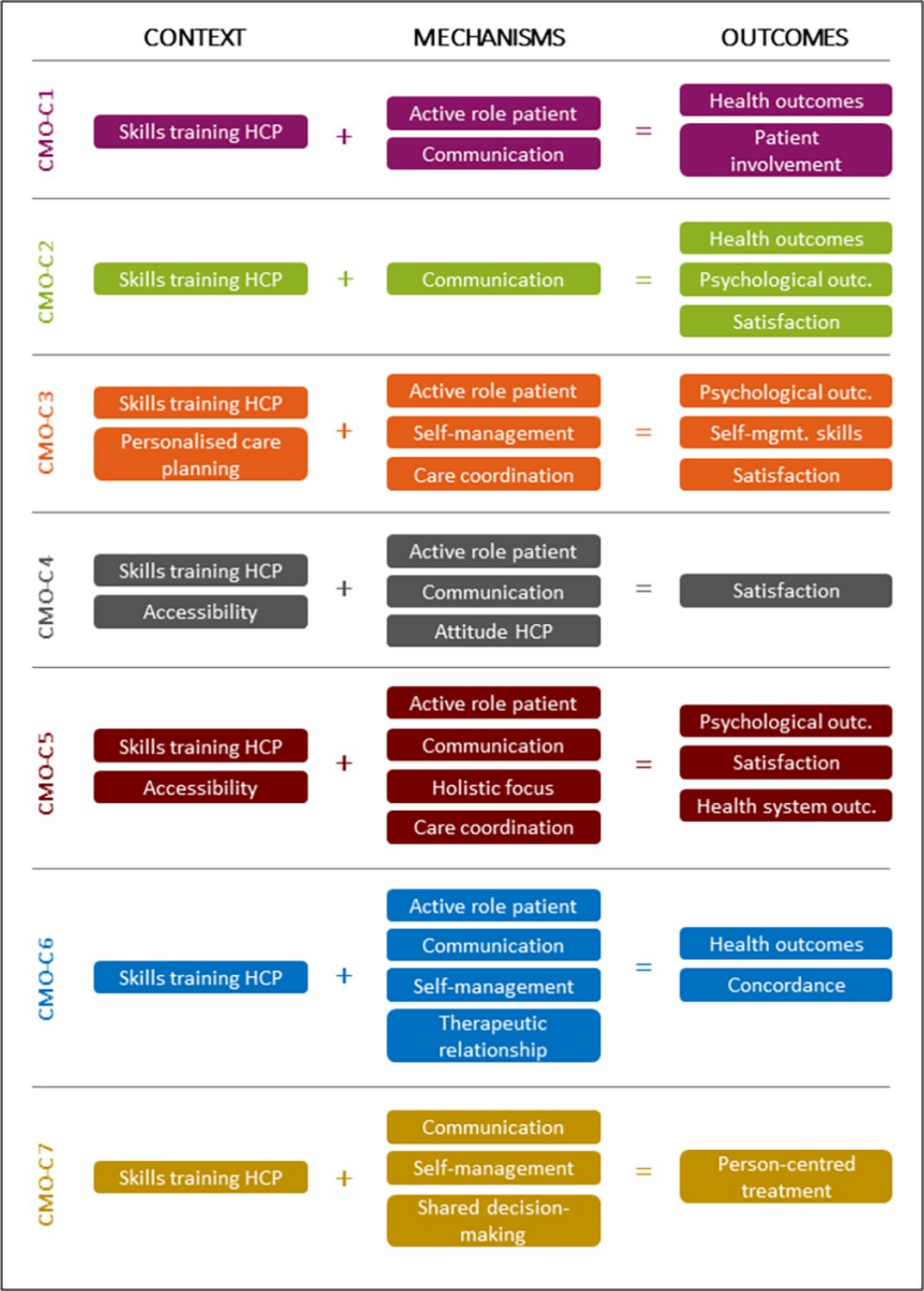
Middle-range PT from the RRR [20].

Healthcare systems are gradually transforming from biomedically-oriented systems towards more person-centred care (PCC) oriented systems [1, 2]. To understand and adequately address a person’s health problem(s) and experience of illness, having a disease-oriented perspective alone is not sufficient [3, 4]. Worldwide, person-centredness has gained more recognition over the years and is considered a core element of high-quality healthcare [5–7]. Driving factors behind this recognition are the growing and changing demand for care, more technological possibilities, and the rising healthcare costs [8]. When PCC addresses also non-medical causes of and solutions for physical distress, it could reduce costs of more expensive (hospital-based) medical specialist care. A core element of PCC is to create a partnership between the healthcare professional and the care recipient, in which the unique needs and beliefs of the latter are the starting point for the provision of care [9]. PCC is considered a core value of primary care [10, 11]. In the Netherlands general practitioners (GPs) have a central role in the healthcare system. As GPs are the first contact point for individuals experiencing health problems and an increasing number of patients with complex care needs ending up in primary care, it is especially important for GPs to provide appropriate support by applying a holistic and person-centred approach that contributes to the overall well-being of individuals [12]. The Dutch healthcare system is recognised for its well-developed primary healthcare [13, 14]. Important elements for this are GPs acting as gatekeepers for specialist care and hence the gradual accessibility of secondary medical specialistic care. The assumption behind this is that a well-functioning primary care setting takes over the care demand as much as possible, which otherwise would end up in the more expensive secondary care. The implementation of practice nurses in Dutch GP practices has increased the interdisciplinary character of care [15]. In addition to the gatekeeping function, empanelment is also considered an important component for building or strengthening primary care [16]. Literature advocating PCC is widespread [17] and the experiences gained with PCC in primary care in the Netherlands are increasingly shared, often in terms of best practices, barriers to implementation and conditions for success [18]. However, despite the conceptual attractiveness of PCC, in daily practice PCC remains poorly understood and implemented [19]. A previously published rapid realist review (RRR) of international literature aimed to provide insight into the question for whom, how and why PCC in primary care does (not) work under what circumstances [20]. The resulting middle-range programme theory (PT) (Fig 1) demonstrated that healthcare providers (HCPs) should be trained and equipped with the knowledge and skills to communicate effectively (i.e., in easy-to-understand words, emphatically, checking whether the patient understands everything, listening attentively) tailored to the wishes, needs and possibilities of the patient, which may lead to higher satisfaction of patients, informal caregivers, and/or healthcare professionals. This way patients will be more involved in their care process and in the shared decision-making process, which may result in improved concordance, and an improved treatment approach. A respectful and empathic attitude of the HCP plays an important role in establishing a strong therapeutic relationship and improved health (system) outcomes. Together with a good accessibility of care for patients, setting up a personalised care planning with all involved parties may positively affect the self-management skills of patients. Good collaboration within the team and between different domains is desirable to ensure good care coordination.

However, since the application of PCC in primary care in the Dutch setting is expected to differ from primary care in other countries, it is deemed relevant to assess the relevance of the obtained items from the international RRR for the Dutch setting. In doing so, the active involvement of experts from the field is of great importance, both for providing input and for translating theoretical insights into suggestions for daily practice [21]. Moreover, PCC should also take into account diversity in age, gender, socio-economic status, education, migration background, (multi)morbidity as well as personal preferences and needs [22]. For example, approximately 25% of the Dutch population has a migration background [23], more than 18% are low-literate [24], and 30% have insufficient or limited health literacy skills [25]. People from these groups often have poorer health, partly because the care provided insufficiently match their needs and possibilities. Existing treatment protocols and standards of care are largely based on scientific evidence usually obtained from study participants outside these groups and therefore do not or only partially apply to these groups [26].

The objective of this study is to validate the items (face validity) resulting from the international RRR for the Dutch setting by assessing consensus on the relevance of the items among different stakeholders.

## Methods

### Patient and public involvement

This study was commissioned by the National Health Care Institute, who, amongst others, encourages good healthcare by helping all parties involved to continually improve healthcare quality. This study is part of a larger study for which a steering committee was established. The ten members of the steering committee were purposively selected based on their expertise in the PCC or primary care field and were primary care practitioners, senior researchers, medical specialists, policy makers, patient’s representatives (specifically concerning patients with limited (health-)literacy and a migrant background) (see Acknowledgements). Several meetings with the steering committee were held during the study (February 2018, December 2018, April 2019, December 2019). These meetings were held with the objective to provide feedback and guidance on the methods, the interpretation of (interim) results, and providing overall advice regarding the research. Stakeholder perspectives were considered when testing and refining the PT derived from the RRR. Members of the steering committee were asked to discuss, and to indicate if the identified items on context, mechanisms and outcomes in the literature match with what they see in Dutch practice.

### Programme theory

One of the key elements in doing realist research is to establish a PT. A PT explains what mechanisms will generate the outcomes and what features of the context will affect whether or not those mechanisms operate [27, 28]. Context items refer to wider external factors, and mechanisms are considered enablers, underlying entities, processes, structures, reasoning, choices, or collective beliefs). The interaction between context and mechanisms lead to outcomes (intended and unintended). In the international RRR we established a middle-range PT (see Introduction and Fig 1), which we aimed to refine based on the findings of this study in the Dutch setting.

### Study design

In this qualitative study, four focus group discussions (FGDs) were held with the objective to encourage group interaction between participants and to explore and clarify individual and shared perspectives [29]. FGD 3 and 4 were combined with a Delphi-study. The four FGDs were held with different stakeholders to validate the findings from the international RRR for the Dutch setting. A FGD lasted approximately 90 minutes. All FGDs were held at a neutral place that participants already knew (i.e., at a research organisation), and where they felt comfortable. Participants of FGD 1 and 2 were patient representatives and patients with limited health literacy skills. Participants of FGD 3 and 4 were various primary care professionals. Due to the different target groups, a target group-specific approach was used. The different approaches are explained in more detail below.

### Recruitment

Participants of FGD 1 and 2 were recruited through purposive sampling. Adult participants were approached using trusted network organisations. These organisations are the Network of Organisations of Older Migrants (NOOM), which focus on diverse groups of migrant older people in the Netherlands, and the ABC foundation, a volunteer organisation for low-literate people throughout the Netherlands. During the recruitment process maximum variation in gender, age, ethnic background, educational level and level of health literacy was aimed to achieve. FGD 1 and 2 were led by a researcher [AA] and another moderator experienced in leading FGDs with people with low (health) literacy skills [NHvR]. FGD 1 and 2 took place in August 2018.

Participants of FGD 3 and 4 were various primary care professionals, members of care organisations, policy makers, and researchers. Participants of FGD 3 and 4 were recruited (purposive sampling) through the expert network of the researchers of this project, aiming for variation in gender, age, professional background, and experience with person-centred care. To be included in the FGD, participants needed to have scientific (research) experience and/or practical work experience in a professional or service organisation regarding person-centred care in primary care. FGD 3 and 4 were led by two researchers [AA and HJMV or MvdM]. FGD 3 and 4 took place in December 2018.

### Data collection

**Fig 2:**
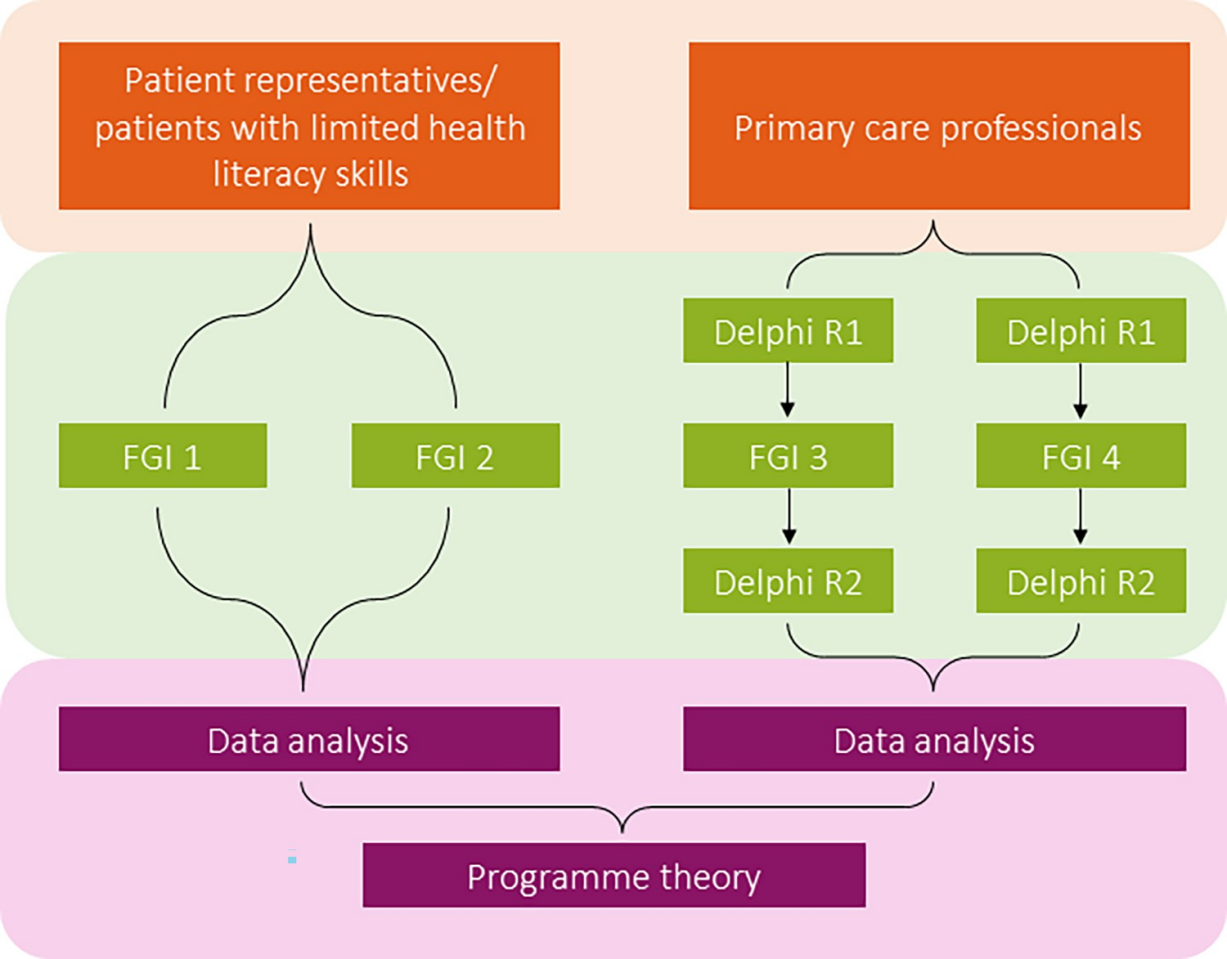
Overview of participants, data collection, and data analysis.

For FGD 1 and 2 an open-ended semi-structured topic guide was used by the moderators, which was compiled based on the context items, mechanism, and outcome variables from the RRR (Fig 1). Only patient-related items were included and were presented in the form of simple formulated questions during the FGDs (Fig 2 and S1 File). Participants could also ask other questions and/or share their own story or experiences. This facilitated the researchers to collect additional data. Participatory learning and action (PLA) techniques were applied to facilitate equal input from participants, thereby stimulating the active participation of participants. PLA is a form of participatory research, which emphasizes the need for stakeholders’ active engagement across the full range of research activities, including data generation and data analysis, and is specifically suitable for meaningful involvement of stakeholders with limited power or skills [30, 31].

Field notes were made during the FGDs. In FGD 3 and 4 validation of the CMO-items by participants took place by means of an e-Delphi questionnaire (S2 File) and a FGD during the second round (Fig 2). The Delphi technique is a widely used research method, which consists of several rounds of data collection to capture and structure the knowledge and opinions of a panel of participants on a topic in which they have expertise [32]. Field notes were made during the FGDs.

#### Delphi round 1

Participants received a web link to an online version of the questionnaire in SurveyMonkey (version 2018). The questionnaire started with an introduction of the study and its objectives, the structure of the questionnaire, and the definitions of the constructs: context, mechanisms, programme-activities, and outcomes. The questionnaire continued with six general questions regarding gender, age, highest level of education, current job position, number of years working within the position, and number of years of experience with PCC. The questionnaire contained another 63 questions related to CMO-data derived from the RRR. Experts were asked to assess the relevance on a 9-point Likert scale (1 = very irrelevant to 9 = very relevant) of PCC-related items in primary care in the Netherlands of context items (n = 30), mechanisms (n = 19), and outcomes (n = 14) identified in the RRR. The questionnaire ended with two open questions, namely possible additions to the stated context items, mechanisms, and/or outcomes based on personal experiences, and participants were asked if they had any additional comments/suggestions about the questionnaire. The answers of the participants were completely anonymised. The respondents were given a total of two weeks to complete the questionnaire.

#### FGD (second round)

Before the second round of the Delphi questionnaire was completed, a FGD was held (Fig 2). The aim of this FGD was to discuss the context items, mechanisms and outcomes for which insufficient consensus/dissensus was found in round 1. During this FGD, the group results from the first Delphi round were provided, including 1) the median assessment results and interquartile range (IQR) on each item), 2) the level of (insufficient) consensus between the participants and, whether consensus was achieved [32, 33]. The IQR is the difference between the 3^rd^ and 1^st^ quartile in which 50% of core values lie [34] and also shows the degree of convergence of the answers [35–38]. The items, for which dissensus was found, were presented and discussed during the FGD to give insight into the level of (dis)agreement between experts in the first round and to generate additional insights about the specific item(s). Providing feedback on the level of group agreement reached, influences achieving the level of consensus subsequently [39]. Misinterpretation on item(s) needed to be clarified.

#### Delphi round 2

An online version of the questionnaire was sent including the context items, mechanisms, and outcomes for which no consensus was found in round one [33]. The questionnaire started with the same general questions as round 1. Then, participants were asked to indicate the degree of relevance of context items, mechanisms and outcomes for PCC in primary care in the Netherlands on the same 9-point Likert scale. At the end of the questionnaire, participants had the possibility to add items that were not included in the questionnaire and could also provide general comments/suggestions on the questionnaire. For round 2, the respondents were given a total of two weeks to complete the questionnaire.

### Data analysis

All FGDs were audio-taped and transcribed verbatim manually. Using thematic analysis techniques [40], text segments were assigned a code if they related to a specific theme/topic, using an inductive, iterative process. Categories with similar content were investigated for inter-relationships, and further refined. Half of the data was coded independently by two researchers [AA, MvdM] to maximise credibility and trustworthiness [40]. Any differences in code application were resolved by discussion with a third researcher [HJMV]. Data were analysed both descriptively and exploratively.

For the Delphi rounds in FGD 3 and 4 a 9-point Likert scale (1 = very irrelevant to 9 = very relevant) was used to indicate the degree of relevance of the CMO-items. To collect data from participants in a most sensitive matter, use was made of a 9-point Likert scale. For analysis, data were recorded into: irrelevant (1–3), equivocal (4–6) and relevant (7–9). Recoding enabled us to assess consensus on these meaningful levels and hence derive recommendations for improvement. To determine the level of consensus within the Delphi panel, many studies use a predetermined level of consensus among the experts [41]. However, the literature does not describe a standard threshold for reaching consensus [42], with thresholds for consensus varying from 55–100% [43]. In this study the level of consensus was 75% or more [42, 44, 45], with the condition that less than 15% of participants scored in the opposite range of that scale namely the 1–3 range [46, 47]. All items with scores in the 4–6 range and without consensus, were presented again to the Delphi panel in round 2. Respondents’ overall consensus on each context, mechanism, and outcome was analysed based on the median of the group’s scores. The analysis was performed in MS Excel 2018.

Consensus on items being found relevant by FGD 1 and 2 and/or FGD 3 and 4, remained part of the PT or were added to the PT. Consensus on items being irrelevant or no consensus on items were removed from the PT.

### Trustworthiness

This study largely complies with the COnsolidated criteria for REporting Qualitative research (COREQ)

Checklist, a checklist for explicit and comprehensive reporting of qualitative studies (in-depth interviews and focus groups) [48]. To increase the credibility of this study multiple FGDs were held, multiple stakeholders’ perspectives were included, and triangulation of data collection methods took place. Regarding transferability, sampling strategies, detailed descriptions of participants, a description of the topic list, and the procedure of methods were included. With respect to confirmability, (interim) results were presented to the commissioner of this study and the steering committee of this study. Regarding dependability, multiple authors independently coded the transcripts, interpretation of the results took place individually by multiple authors, and participants quotations were included to accurately report their perspectives.

### Ethics

As this study does not involve patients or study subjects, according to the Dutch Medical Research in Human Subjects Act (WMO) in the Netherlands, an ethical approval was not needed. However, all participants provided their (verbal) consent and participation in the survey was anonymous.

## Results

### FGD 1 and 2 with patient representatives

FGD 1 and 2 consisted of a total of 14 participants. In Table 1 the participants’ characteristics are shown. Participants who were not originally born in the Netherlands have been in the Netherlands for on average of 44 years (SD: 11.4 years).

**Table 1:** Characteristics of participants.

Characteristic		FGD 1 and 2 (n = 14)	FGD 3 and 4 (n = 11)
Gender (%)	Female	36	45
	Male	64	55
Age (years)	Average (SD)	66 (9.7)	50.1 (10.2)
Highest level of education (%)^#^	Elementary education	57	-
	Intermediate vocational education	21	-
	Bachelor	7	27
	Master	14	45
	PhD	-	27
Background (%)	Research/academic	-	36
	Healthcare provider	-	36
	Other	100*	27^
Years of experience	Average (SD)	N/A	13.6 (11.1)

# Basic education also includes special basis education (e.g., visual/hearing impaired, disabled, chronically ill)

* ‘Other’ includes e.g., a chef/cook, retirees, a stay-at-home mom, IT-teacher

^ ‘Other’ includes e.g. (policy) advisors, managers/project leaders

All context items, mechanisms, and outcomes presented to participants were found relevant for PCC in primary care in the Netherlands. This concerns the context items: patients having social support (networks), a good collaboration between HCPs, patient education being provided, sufficient time during consultation, setting up a personalised care planning, and making use of e-health options.

The mechanisms deemed relevant for PCC in primary care in the Netherlands are HCPs providing effective communication (including listen attentively), HCPs having a holistic approach, HCPs showing respect and having an open, friendly, and empathic attitude, patients having an active role in their care process, establishing a therapeutic relationship, self-management support, and shared decision-making. The outcomes considered relevant concerned health outcomes, patient involvement, satisfaction of the patient, therapy concordance, self-management skills, and an improved treatment approach.

On the items below participants had additional comments next to them being considered relevant. The participants reflected on these items based on their own experience, indicating that they are relevant for PCC in primary care, but not always carried out properly in practice.

#### Communication

According to the participants, HCPs did not (yet) adapt their communication sufficiently to the needs and wishes of the patients. Participants stated that *“in the communication by the care provider more attention should be paid to diversity”* (P1 and P2). One participant expressed that *“communication is extremely important when you visit the GP*. *Often older migrants cannot communicate well in Dutch*, *but they do know what they want to ask in their own language*. *They often bring their son or daughter to the GP together with them to ask questions [related to medical health of patient]”* (P1). In addition, the use of aids (pictures, attributes, etc.) during the consultation could support communication, which is currently very limited done. Also, patients often had difficulties understanding health information and medical terms, while most of them did not indicate this. This is particularly the case for low-literate people and migrants, who had difficulty with the (Dutch) language and were therefore limited in their communication options. One participant mentioned that *"people still don’t have the guts to say they are illiterate*, *and that’s just because of the shame associated with it"* (P3). Reinforcing patients’ language skills and using interpreters can improve communication.

#### Consultation time

An important barrier of PCC in primary care according to the participants was the consultation time with the GP, which is too short to actually explain their problem. A participant mentioned that: *“In my own GP practice*, *I am experiencing the third generation of GPs*, *I noticed that doctors have less and less time*. *The consultation really just takes 10 minutes*, *so you can just ask one question*. *If you have more questions and your time is up*, *you will be cut off*. *It becomes very clear that there is no time left”* (P4). Patients often felt unheard or misunderstood, because there was insufficient time during the consultation to discuss all relevant matters or to explain everything properly. As a result, the HCP was also unable to provide adequate support based on the patient’s context and to discuss any underlying problems. Participants said: *“I would like that he [the GP] gives extra time to people who have difficulties with reading and writing*. *He [the GP] has knowledge in the medical field*, *but he should also know which patient have difficulties with reading and writing*. *Also*, *it should be pointed out what the rules and regulations are here in the Netherlands compared to other countries [regarding time]”* (P5). Patients making a double appointment with the GP could be helpful. Moreover, patients at home writing down points to discuss as preparation of the consultation could contribute to a more efficient use of consultation time. One participant stated that *“healthcare is commercialising in such a way that everything is expressed in Euros*. *The GP would like to take half an hour herself [for the consultation]*, *but the health insurer*, *which is focused on the money*, *plays a very important role here*. *And it’s getting worse*, *I feel*. *Sufficient time and attention for the patient are the building blocks of a relationship of trust*, *and this is at odds with the available time"* (P4).

#### Shared decision-making

Participants experienced that shared decision-making in practice was not conducted properly. Partly because of the short consultation time, the pros and cons of different treatment options were not always explained well by the HCP. Some participants stated that due to insufficient insight of patients into the disease and treatment options, as well as the expectation that the HCP is the expert in the medical field, this resulted in both parties being reluctant to make shared decisions. Therefore, the choice of HCP often played a decisive role. The wishes and preferences of the patient often remained underexposed. Overall, participants mentioned that *“I really like it when a GP asks you if you want to do something [which is part of care process] and whether you agree [with a treatment plan]”* (P6).

#### Collaboration between HCPs

The collaboration between HCPs (e.g., between practice nurse and GP or HCPs between primary and secondary care) could be improved. Participants often experienced that the different HCPs involved in the care process were not always well informed. As a result, patients often had to repeat their story, at the expense of the limited time available. For example, (electronic) information transfer often fell short and relevant (medical) documents were insufficiently shared. The HCPs involved also often gave different advices, which led to confusion among patients. Better coordination between HCPs of the agreements and advices made, is necessary to provide PCC.

#### Active role patient

In certain groups, such as people with low health literacy skills, patients often lacked confidence to ask questions to the HCPs and take an active role for the benefit of their health. This was partly because patients assigned a high status to the GP and placed him/her on a pedestal. These patients often did not want to bother the GP with their questions. In addition, they did not indicate by themselves that they had low (health) literacy skills because of past unfortunate events (e.g., bullying, bad experiences with HCPs ‘not knowing who the patient is’). The patient was also rarely asked by the HCP whether they had low (health) literacy skills, with the result that the HCP had insufficient knowledge about the patient’s background. As one participant stated: *“it would be good if the GP knew the background of the patient and what to consider*. *It is very important that the doctor knows what is going on behind the person in front of him/her”* (P7). Solutions for patients having an active role could be to schedule an intake interview for every new patient in the practice; inform other involved HCPs of important characteristics of the patient (e.g., low literacy); give sufficient room to patients to ask questions, check whether patients have asked all their questions and whether they have understood the answers. On the other hand, patients can go into the consultation better prepared by writing down their discussion points and questions in advance.

### FGD 3 and 4 with care professionals

A total of 18 experts received the invitation to participate in the FGDs, of which eleven experts agreed. In Table 1 the characteristics of the participants are shown.

#### Quantitative description of consensus level

**Fig 3:**
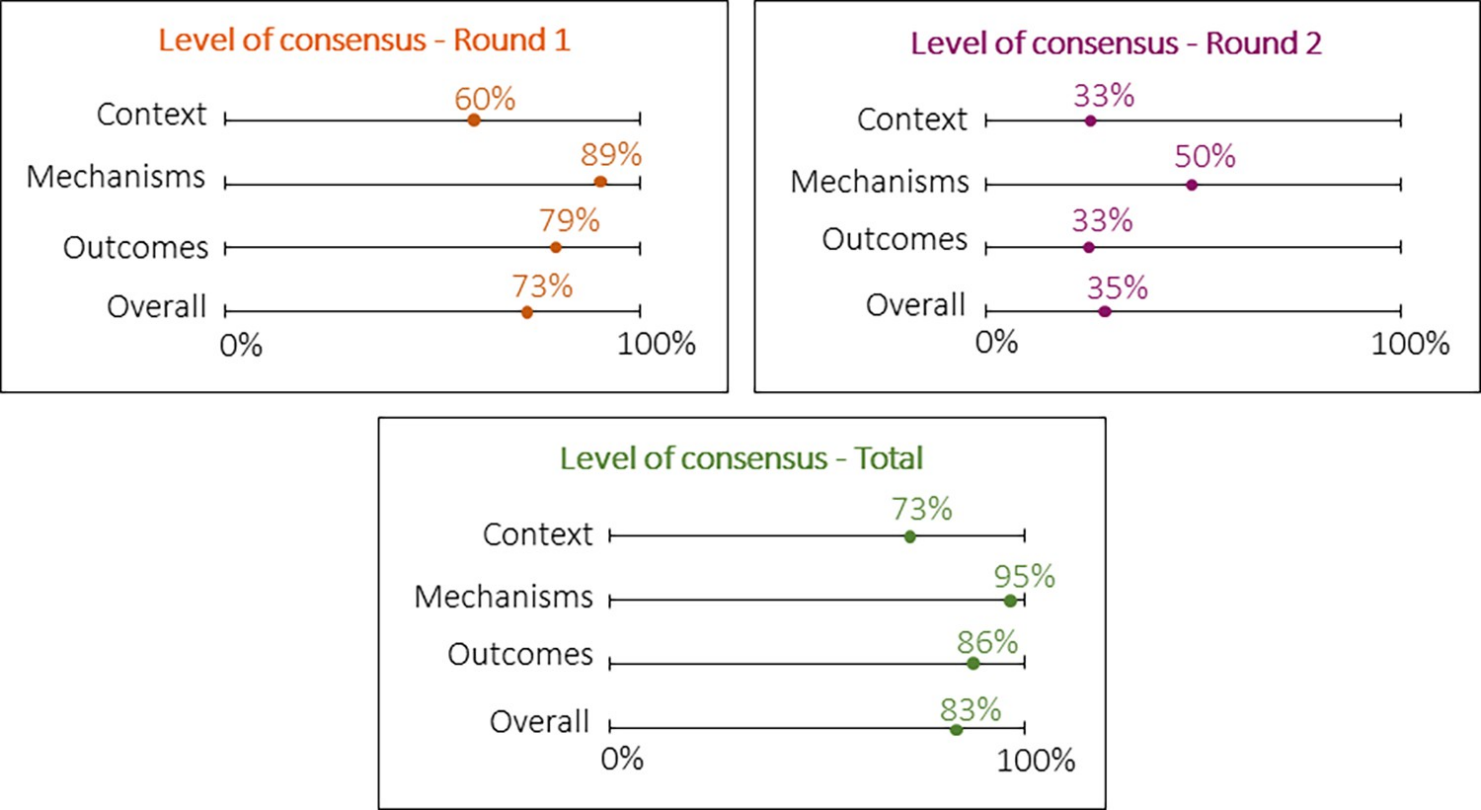
Level of consensus found in round 1 and 2.

In round 1 consensus was achieved for 46 items out of a total 63 items (73%) among experts. All items were found relevant for the Dutch setting with the overall median lying in the 7–9 range. On 18 out of the 30 context items consensus was found (60%), 17 out of 19 mechanisms (89%), and 11 out of 14 outcomes (79%) (Fig 3). On 17 items dissensus was found with a panel median in the 4–6 point range (3 items) and 7–9 point range (14 items). These items were included in round 2.

In the second round, consensus was achieved among experts for 6 out of 17 items (35%), of which 4 out of 12 context items (33%), 1 out of 2 mechanisms (50%), and 1 out of 3 outcomes (33%). The overall median was in the 7–9 range. For 11 items, the relevance remained undecided. The overall median was in the 4–6 range (5 items) and in the 7–9 range (4 items), 2 items equally fell in the 4–6 range and 7–9 range. After both rounds, for 52 items out of 63 items (83%) consensus was found with all items being considered relevant.

#### Qualitative description of context items, mechanisms, and outcomes

The outcomes on every context item, mechanism, and outcome of the first and second Delphi round are shown in S3 and S4 Files respectively. The items from round 1 that were found to be equivocal, were included in the second round.

### Consensus

#### Context items

Based on both rounds, context items that were considered relevant for PCC in primary care in the Netherlands on macro-level were shifting the focus from a disease- and complaint-oriented approach to a more holistic approach, using evidence-based guidelines, foreseeing in sufficient capacity and time for patients during consultation, offering (more) space and resources to HCPs to experiment, and having flexible payment systems. Participants believed that *“experimenting in its broadest sense should be taken into account to improve PCC towards patients”* (P10, P13, P18). *“For example*, *if you have patients with a chronic conditions and you want them to take more control of their health themselves*, *and as a care provider you have learned a new conversation technique to be applied during consultation in which you approach the person openly and let him/her decide for themselves what they want to change [in their care process]*, *then you have to have the space to try out the new technique*, *practice with it*, *and to improve it”* (P16).

On an organisational (meso) level, experts found that improving accessibility (e.g., to healthcare organisations, to documents, recorded consultations), having a good collaboration between HCPs and having a shared vision, having a supportive policy in place which strengthens the quality of PCC especially concerning low health literacy, and better integration between information and communications technology (ICT) systems are relevant items. Of the latter a participant mentioned: *“Better integration between ICT systems promotes cooperation*, *care is then better coordinated and it becomes more person-centred*. *Now everyone works according their own way”* (P12).

On an individual (micro) level HCPs having PCC skills (e.g., regarding communication, shared decision-making, providing culturally sensitive care) possibly through training or acquired during their medical education was found relevant. In addition, HCPs providing patient education, patients having social support (networks), and patients being involved in organising care was considered relevant.

A participant mentioned that *“HCPs setting goals and making action plans is also very relevant*, *because often patients don’t know this by themselves*. *They often have questions during the consultation*, *and when the care provider reaches the bottom layer of those questions*, *you discover why the patient finds that important*. *Also*, *other things that are important for the patient emerge”* (P10).

#### Mechanisms

On meso-level experts found a focus on care coordination and achieving effective collaboration between patient and HCP(s) relevant. On micro-level, it is key that HCPs provide effective communication (e.g., simplifying treatment strategies and information for patients, encouraging patients to ask questions), have an open and empathic attitude, are aware of the patient’s social circumstances, have a holistic focus, respecting the wishes and preferences of patients, applying shared decision-making together with patients, provide self-management support, and establishing a therapeutic relationship. Also, the involvement of patients and their family/informal caregivers in the care process was found relevant.

#### Outcomes

The following outcomes were considered relevant for PCC in primary care: an improved treatment approach with a more accurate intensity of support provided, higher therapy concordance, increased patient involvement, improved (psychological) health outcomes, improved health-related quality of life (HRQoL), higher satisfaction of patient, informal caregiver and/or HCP(s), improved relationship between patient and HCP(s), more accessible care, higher quality of care, and a higher cost-effectiveness of healthcare. One participant mentioned: *“Intensity of the support provided by the HCP is very important as an outcome*. *You could consider it as a success factor of PCC*, *it is tailored support to the patient”* (P12).

### Dissensus

#### Context items

After two rounds, a lack of agreement on the relevance of some items for PCC in primary care in the Netherlands was observed, such as the application and efficient use of ICT and e-health initiatives. *“The information in e-health applications needs to be in line with what the healthcare provider says*. *Only if the information is in line and explained well*, *it will reinforce each other*, *otherwise it will lose its function*.*”* (P13) *“E-health applications may not work for low-literate people or non-native speakers*. *Moreover*, *there are also people that are digitally illiterate”* (P14).

There was also dissensus on the item having sufficient male and female HCPs per practice, as participants found that *“there are people who would like to have a male or a female care provider*, *it’s nice that people have that choice*. *But whether you choose a male or female doctor*, *they both have to provide PCC*, *regardless of their gender”* (P15).

Some participants believed that providing better administrative support for HCPs might positively influence PCC, but is not considered relevant to provide PCC. *“Providing better administrative support for caregivers can reduce administrative barriers to increase working in a person-centred way*. *The [consultation] time you can spend on a patient is already limited*, *so if you can spend less time on administrative things such as electronically saving or capturing what has been discussed with the patient such as setting the goals*, *you have more time to provide PCC to the patient*. *But it is not a precondition to provide good PCC and therefore*, *not relevant”* (P16).

Regarding the item preparation of consultation by patient it was mentioned that *“the preparation of a consultation by the patient is not by definition relevant for the provision of person-centred care by the care provider”* (P9). *“It is nice if a patient prepares a consultation*, *it can be very helpful*. *The question is also whether each patient can prepare the consultation*, *whether he/she is competent enough to do so*. *Someone who actively thinks about his/her health makes the conversation easier*, *but it is not a condition for the provision of PCC*, *that is the task of the care provider”* (P8).

About the item patients having a high/low socioeconomic status (SES), some mentioned that *“having a high or low SES is not relevant for providing PCC*. *Most of the time it does require more effort to provide PCC to people with a low SES*. *But providing care to people with a high SES*, *such as expats*, *can also be challenging*, *as they are not familiar with the systems [in the country]*, *but are highly educated at the same time*. *SES is not decisive for PCC”* (P12, P15).

Dissensus was also found on the items setting up a personalised care planning and, HCPs stimulating patient empowerment.

#### Mechanisms

There was no agreement on the relevance concerning HCPs stimulating self-monitoring by patients. It was mentioned that *“It is important that the patient can monitor his own medical condition*. *However*, *a person with low health literacy skills with for example severe rheumatism may need someone else to monitor him/her*. *Stimulating by the care provider is important*, *but you have to take into account what someone is able to do*. *I don’t think everyone can and will monitor their own health*. *It is beneficial for those who can”* (P11).

#### Outcomes

No consensus was found on the items self-management skills of patients and health system outcomes (reduced use of healthcare system, less referrals, less follow-up examinations, reduced emergency department visits, reduced hospital (re)admissions) for PCC in primary care in the Netherlands.

### Additional items

In addition to the items identified in the literature, the participants stated several other items, such as caregivers having more pleasure in their job as an outcome. To enhance (the focus on) PCC in primary care for low health literacy skills groups, the expertise of professionals who are familiar with working and treating these groups from diverse backgrounds could be used (i.e., peer education). Another item mentioned was that when involving patients in their care process, the responsibilities of the patient and HCP need to be clearly defined.

### Refined programme theory

**Fig 4:**
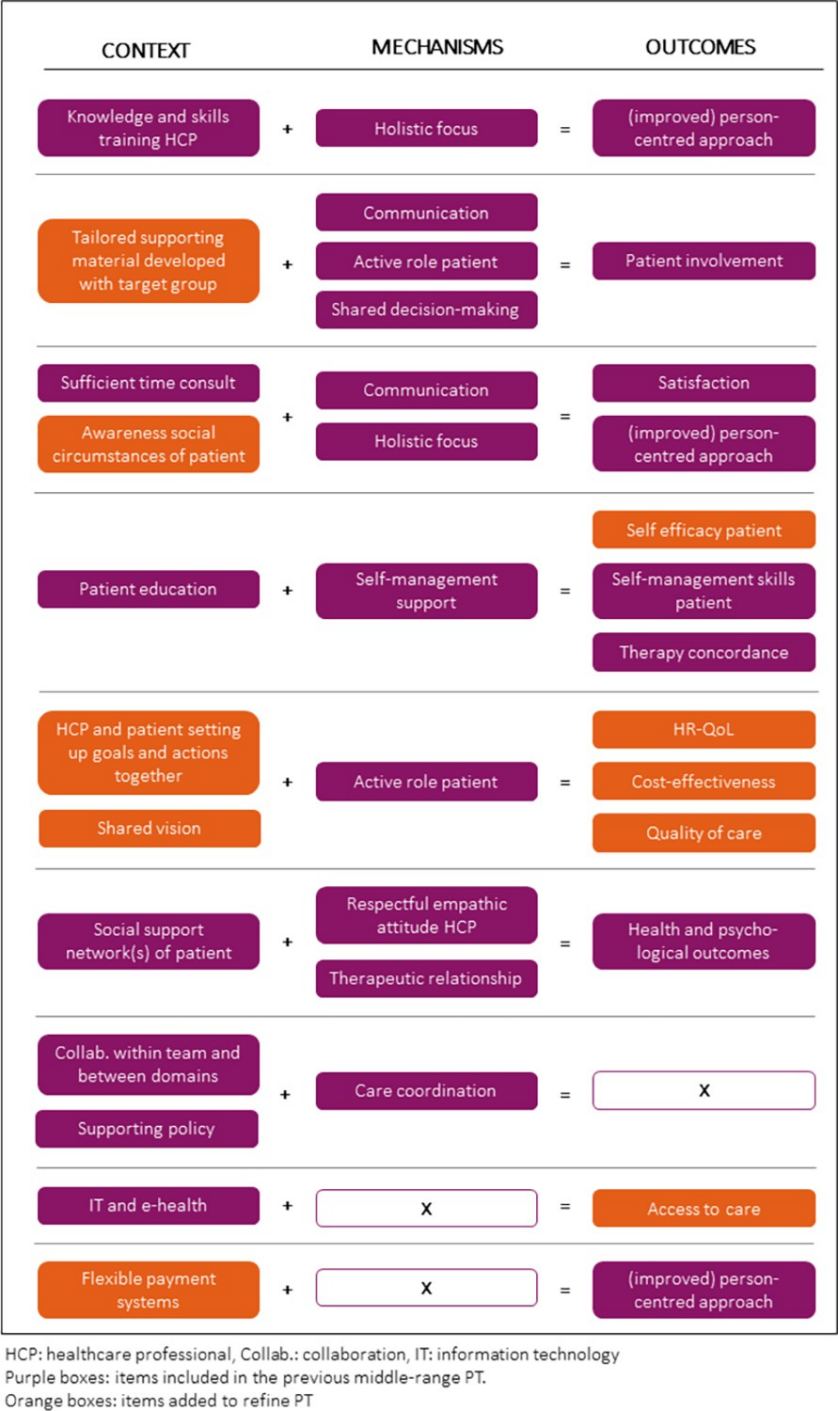
Refined PT by FGDs.

Based on the results of the FGDs, the middle-range PT derived from the international RRR has been refined for the Dutch setting (Fig 4). In this refined PT the context items (C), mechanisms (M), and outcomes (O) that have been added, are underlined. The non-underlined items were already included in the middle-range PT.

The refined PT demonstrated that to provide a better intensity of support to the patient (O) and optimally align care to the patient (O), it is necessary that HCPs are equipped with the knowledge and skills and are trained and educated (C) to have a holistic focus (M) taking into account the diversity aspect (C), instead of a biomedical, disease-oriented approach (C). Communication (M) tailored to the needs and health literacy skills of the patient plays an important role, just as tailor-made supporting material (C) being available for patients. By developing these together with the target group (C), it is more likely these will match the target group and contribute to realising a more active role of the patient (and their families) in the care process (M, O), and in the shared decision-making process (M). To communicate effectively (M), HCPs should be provided with sufficient time and space (C), also to become aware of the patient’s (social) circumstances (C), discuss the wishes and preferences of patients (M), and work in a culturally competent way (C). As a result, a higher satisfaction of patient, informal caregivers and/or HCP(s) (O) can be achieved and the PCC treatment approach (O) can be improved. If several HCPs are involved in the care process, good collaboration within the team (C) and between different domains (C) is desirable to ensure good care coordination (M). These elements can be stimulated by including them in the policy of (care) organisations, wherein attention is also paid to people with low health literacy skills (C). HCPs having an open, respectful, and empathic attitude (M) plays an important role in establishing a strong therapeutic relationship (M). Patient’s social support networks (C) also help to improve the patients’ (psychological) health (O). In addition, better integration between ICT systems (C), offering e-health options and access to documents, recorded consultations (C), play a key role in a more accessible care (O). Flexible payment models (C) could facilitate PCC in primary care (O). Next to providing patient education (C), HCPs should provide self-management support to patients (M), stimulating patient’s self-management skills (O), self-efficacy (O) and therapy concordance (O). When goals and action plans are set up together during personalised care planning (C), HCPs and patients have a shared vision (C), the patient has more confidence to ask questions (C) about the treatment (possibilities), and has more insight into the importance of his/her treatment (M), this may lead to improved HRQoL (O). On the long-term, higher cost-effectiveness of healthcare (O) and a higher quality of care (O) can be accomplished.

## Discussion

### Principal findings

In this study the middle-range PT from the international RRR was refined for PCC in primary care in the Netherlands by assessing the level of consensus on the relevance of items derived from the RRR by means of FGDs and a Delphi-panel.

Based on the FGDs, several items have been added to refine the PT. The context items that were added concern HCPs being aware of the patient’s (social) circumstances, working in a culturally competent way, HCPs and patients having a shared vision and setting up goals and action plans together, patients having more confidence to ask questions, providing tailor-made supporting material, developing supporting material and tools together with the target group, a better integration between ICT systems, providing patient access to documents and recorded consultations, and flexible payment models being in place. No mechanisms were added. Outcomes that were added include better alignment of care to the patient, having accessible care, improving the patient’s self-efficacy, improving HRQoL, higher cost-effectiveness of healthcare, and a higher quality of care. One item was excluded from the middle-range PT to refine the PT as not all FGDs found this item relevant for PCC in primary care in the Dutch setting, namely improved health system outcomes (outcome).

This study makes clear that sufficient attention needs to be paid to the complex interplay of the context items, mechanisms and outcomes concerning PCC in primary care in the Netherlands. Bypassing this complexity will most likely not lead to the desired effectiveness of PCC in primary care. The use of all items in their mutual coherence is necessary to truly realise PCC.

### Strengths and limitations

One of the strengths of this study is the use of the combination of FGDs and the Delphi method. The participation of both–the often thought of as hard to reach—patients with low (health) literacy levels and primary care professionals increase the face validity of the results of this study. A possible limitation concerns the limited number of FGDs.It is suggested to conduct two to three FGDs to capture 80% of themes, and three to six groups for 90% of themes [49]. However, data saturation seemed to be reached as in the second and fourth FGD no new items were mentioned than in the first and third FGD. Also, there were no specific inclusion criteria for participants of FGD 1 and 2. These participants were recruited through convenience sampling. A third limitation to be considered is that the group moderators of FGD 3 and 4 were not impartial to the study. Nevertheless, they only moderated the discussion and did not share their own opinions.

### Comparison to previous studies

Consistent with our refined PT, studies have found that in order to deliver effective PCC the patient wishes, needs, and abilities need to be taken into account to align care to the patient [50, 51]. Also, HCPs should stimulate patients to set and achieve their own treatment goals, and access to care should be optimised [50]. The importance of providing tailored supporting materials, culturally competent working, and self-efficacy of the patient has also been reported [51–53]. Individualised care plans, physical comfort at GP practice, and providing patients emotional support were also mentioned, but not found in our study [50].

### Implications for practice and research

Given the complexity of the interplay of all items, it is recommended *for healthcare organisations* to develop and implement an all-encompassing approach and to divide the approach into phases, to make it manageable. During the first phase (initiation) HCPs need to acquire relevant knowledge and skills through education and training. Patients need to be aware of their role in their care process and that they have social support networks. In the second phase (decision & adoption) adjustments regarding the healthcare system, policy-making, financing issues, integration between ICT systems, and creating sufficient experimental space, time and resources are made concrete. In the third phase (execution) the focus is on the implementation of a good collaboration between HCPs, the provision of self-management support, patient education, shared decision-making, whereby information and communication should be simplified. In the fourth phase (monitoring & evaluation) it is necessary to gain insight into (unexpected) problems and challenges, to find out to what extent the intended results/effectiveness are being achieved and to meet the needs for resources. With respect to further research, it is recommended to assess how and to what extent the items have been collectively implemented and to evaluate how effective PCC is in practice, for whom, how and why. Also, items on which dissensus was found need to further examined why they were found irrelevant for the Dutch setting. Our understanding of PCC is likely to increase (faster) when applying realist research iteratively and in different settings.

## Conclusion

This study shows that for PCC to be effective in primary care, the complex interplay of context, mechanisms, and outcomes deemed relevant to a setting must be met. Added items to refine the PT for the Dutch primary care setting indicated that to optimally align care to the patient, next to tailored communication, also tailored supporting material that is developed together with the target group is key. HCPs and patients need to have a shared vision and set up goals and action plans together. HCPs should stimulate patient’s self-efficacy, need to be aware of the patient’s (social) circumstances and work in a culturally sensitive way. Better integration between ICT-systems, flexible payment models, and patients access to documents, recorded consultations should be in place. On the long-term higher cost-effectiveness and a higher quality of healthcare can be realised when sufficient attention is paid to the interplay of relevant context items, mechanisms and outcomes.

## Supporting information

S1 File Topic guide for FGD 1 and 2. (PDF)

S2 File Delphi questionnaire for FGD 3 and 4. (PDF)

S3 File Results Delphi round 1. (PDF)

S4 File Results Delphi round 2. (PDF)
