# Supplementary material for: Person-centred care in the Dutch primary care setting: Refinement of middle-range theory by patients and professionals
Source: PLoS One. 2023 Mar 9;18(3):e0282802. doi: 10.1371/journal.pone.0282802 (PMC9997984; doi:10.1371/journal.pone.0282802)
Supplement: S1 File — (PDF) [file pone.0282802.s001.pdf]

Supplementary file 1. Topic guide for FGD 1 and 2

1.	Communication/relationship with healthcare provider
	- What do you like about your GP? How would you like your GP to act?<br>- Do you think that your GP pays enough attention to you during the appointments/consultations, for example do you have the feeling that the doctor listens to you and responds to what you tell the doctor? If not, what could be better?<br>- Do you think the doctor has enough time during the appointments? If not, do you think more time is needed? Why do you need more time?
2.	Empathy by healthcare provider/empathy in communication
	- Do you think that during the appointments your GP 1) sympathizes/thinks along with you, 2) understands your problem or question?
3.	Good communication/understandable information material, and simplified information
	- Does the GP adapt his communication sufficiently to you? Do you understand what your doctor means?<br>- Do you like the conversation you have with your GP during the appointments?<br>- Does the doctor use things like pictures or information on the computer to explain things?
4.	Building a confidential relationship/partnership between patient and healthcare provider
	- Do you feel comfortable with your GP?<br>- Can you tell everything to your GP? Do you feel safe with your GP?<br>- Do you have self-confidence to ask all questions to the doctor?<br>- Do you think the relationship/bond with your GP is important?<br>- Do you trust your GP?
5.	Transparancy
	- Do you feel that your GP tells you everything about your problem/situation? That he/she is open?<br>- Or sometimes that he/she doesn't tell you everything? When did this happen?
6.	Awareness of patient’s background
	- Does the GP know your background? Does he/she know that you are low-litterate?<br>- When you first visited the GP, were you told something about what the GP does?<br>- Was something told about how health care works in the Netherlands? For example, information about health insurance with basic package, supplementary package, and deductible? If so, what was said?
7.	Personalized care plan
	- Do you prepare for an appointment with the doctor? And how do you do that? Do you write things down? Are you looking for things on the computer/the Internet?<br>- Is during the appointments discussed what should be done/what the treatment entails and what the result should be (setting goals)? Was this written down?<br>- And was this discussed (assessed) again at the end?<br>- Did you need more information about how to make appointments?
8.	Motivation/stimulation of self-reliance/self-management (support)
	- If you have to do something at home for your medical condition, such as regularly checking your blood sugar, does your GP feel that this is important?<br>- Does your GP tell you what you should do yourself to cope with the disease? So, for example, a good explanation of what to do, why, how, when, and how often?<br>- And does he/she tell you how long you should continue with this and why this is important?
9.	Shared-decision making
	- Does the doctor tell you the different options/choices you have?

	- If decisions have to be made about, for example, a treatment, does the GP do this alone or do you do it together?
10.	Respecting preferences and wishes of patients and involvment family
	- For example, if you wanted or would prefer a certain treatment, will your GP listen?<br>- If you had a particular wish because of culture or religion, did your GP approve?
11.	Regular visits
	- Do you regularly visit the doctor? How often is that? Do you like it better if you visite more often? Do you trust your GP more?
12.	Use of tools and technology
	- Are other things or tools being used to help you with your medical condition? To help you understand your medical conditions or treatment? For example, a special phone number that you can use if you have any questions? Or something on the computer?
13.	Physical, geographical, financial, social, and cultural accessibility to care
	- Are you have pain when you go to the doctor? (physical)<br>- Is it easy to get to the doctor? Walking, cycling, or by car? (geographical)<br>- Do you ever think about the costs if you have to go to the doctor? Or not because you think the costs will be high? (financial)<br>- Are there any social or cultural aspects that play a role when visiting the doctor?
14.	Involvement of patients in the development of new instruments/including patient experiences in setting up care
	- When something new is developed, for example a booklet, a folder (or a new model/instrument), are you asked what is important?<br>- And is it asked what would be helpful when developing new tools/instruments/booklets?
15.	Higher patient satisfaction (outcome)
	- If the GP gives you more attention and asks for more personal things, are you more satisfied with the appointment?
16.	Better health outcomes (outcome)
	- Has your health improved if your GP pays more attention and asks for personal things? So, for example, better sugar values?
