# Supplementary material for: Person-centred care in the Dutch primary care setting: Refinement of middle-range theory by patients and professionals
Source: PLoS One. 2023 Mar 9;18(3):e0282802. doi: 10.1371/journal.pone.0282802 (PMC9997984; doi:10.1371/journal.pone.0282802)
Supplement: S2 File — (PDF) [file pone.0282802.s002.pdf]

Supplementary file 2. Delphi questionnaire for FGD 3 and 4

Title

Person-centred care in primary care: relevance of context, mechanisms and outcomes

Introduction

This questionnaire is part of the study 'Evaluation of person-centred care in primary care'. The research is financed by the National Health Care Institute (ZIN) and carried out by Pharos and Panaxea.

The study focuses on the question ‘for whom, how and why does PCC in primary care (not) work and under what circumstances? The core of PCC is that the care provider does not focus on the complaint or medical condition, but on the person presenting the complaint. The treatment does not focus on the health problem, but on the way in which the patient views life and deals with the problem himself. PCC refers to care that is tailored to the characteristics, the environment and the needs of each patient. Diversity, or differences between patients may exist in age, gender, socio-economic status, education, migration background, and presence of disease(s). Diversity between patients can also be reflected in differences in personal preferences and care needs.

The study consists of three phases. In the first phase of the study, a literature study was conducted, in which information was collected about how and in what circumstances PCC results in outcomes. These findings from the literature review are categorised using three constructs: (1) context, (2) mechanisms and (3) outcomes.

- *Context* refers to the wider external conditions necessary for PCC and the mechanisms to result in outcomes;
- *Mechanisms* are the processes/triggers that arise when PCC results in outcomes under the right conditions;
- *Outcomes* are the results that arise from the interplay of PCC and the mechanisms in a certain context.

To get insight into the degree of relevance of the context items, mechanisms and outcomes identified in the literature, the input of stakeholders is required (phase 2). Therefore, we kindly request you to assess the context items, mechanisms and outcomes of PCC for their relevance in primary care in the Netherlands. Your assessment scores will be included in the survey anonymously.

Questionnaire

The questionnaire starts with some general questions. Then, we will ask you to indicate the degree of relevance of context items, mechanisms and outcomes for PCC in primary health care in the Netherlands. The survey ends with a few open questions, with room for any additional items. We kindly request you to answer all questions.

General questions

- What is your gender? [Male/Female]
- What is your age?
- What is your highest level of education achieved? [none, high school, bachelor's degree, master's degree, PhD]
- What is your current position?
- How many years have you been working in this position?
- How many years of experience do you have with person-centred care?

Context items, mechanisms, and outcomes

In the next section, rate the relevance of each item by checking the box that best fits your answer. There are no right or wrong answers.

[Options: Highly irrelevant, Irrelevant, Fairly irrelevant, Somewhat irrelevant, Neither irrelevant nor relevant, Somewhat relevant, Fairly relevant, Relevant, Highly relevant]

Context

1. Setting up a personalised care planning
2. Preparation of consultation by patient
3. HCPs setting goals, making action plans, coordinating, supporting and assessing care process of patients
4. Training/educating young HCPs during medical education on important aspects of person-centred care
5. HCPs having the right skills (e.g., regarding communication, shared decision-making, providing culturally sensitive care)
6. HCPs having the right knowledge about the epidemiology and the treatment effects in different ethnic groups

7. Having better patient access to documents, recorded consultations (notes, etc.)
8. Improving the accessibility of healthcare organisations
9. Supporting better integration between ICT systems
10. Efficient use of information technology (IT)
11. Applying IT- and e-health initiatives
12. Foresee in the required capacity (time, staff, resources)
13. Having sufficient male and female HCPs per practice
14. Offering (more) space and resources to HCPs to experiment
15. Patients having social support (networks)
16. Having structural attention for low health skills/person-centered care in the policy of the organization
17. Strengthening the quality of care through supportive health policy
18. Aligning healthcare purchasing to local needs/policy
19. Providing patient education
20. HCPs stimulating patient empowerment
21. Having a good collaboration between HCPs/strong team
22. Actively involving patients and patient experiences when designing care (processes)
23. Involving patients in the development of new instruments (tools, step-by-step plan, booklets)
24. Patients having a high/low socioeconomic status
25. Providing better administrative support for HCPs
26. HCPs having a shared vision
27. Using evidence-based guidelines
28. Shifting the focus from a disease- and complaint-oriented approach
29. Foreseeing in sufficient time for patients during consultation
30. Flexible payment systems

<u>Mechanisms</u>

31. HCPs providing effective communication
32. Simplifying treatment strategies and information for patients
33. Investing in understandable information material
34. Encouraging patients to ask questions to HCP(s)/patients having the confidence to ask questions
35. Involving family and informal caregivers in the care process
36. Patients having an active role in their care process

37. Stimulating patient's self-efficacy
38. HCPs promoting involvement, support and reinforcement of patients
39. Providing self-management support
40. Focus on care coordination
41. Establishing a therapeutic relationship
42. Achieving effective collaboration between patient and HCP(s)
43. HCPs having an open and empathic attitude
44. HCPs respecting the wishes and preferences of patients
45. HCPs applying shared decision-making together with patients
46. Have a holistic focus
47. HCPs who are aware of the patient's social circumstances
48. HCPs working in a culturally competent way
49. Stimulating self-monitoring by patient

Outcomes

50. Higher therapy adherence
51. Improved patient-centred treatment/approach
52. Improved intensity of support provided
53. Improved health-related quality of life(HRQoL)
54. Improved self-management skills of patients
55. Higher satisfaction of patient, informal caregiver and/or HCP(s)
56. Improved health outcomes
57. Improved health system outcomes (reduced use of healthcare system, less referrals, less follow up examinations, reduced emergency department visits, reduced hospital (re)admissions)
58. Increased patient involvement
59. Higher cost-effectiveness of healthcare
60. Higher quality of care
61. More accessible care
62. Improved relationship between patient and HCP(s)
63. Improved psychological health outcomes

Open questions

- Do you have additional items to the aforementioned context items, mechanisms, and/or outcomes based on your own experience(s)?
- Do you have general feedback or comments about the questionnaire?

Closing

This is the end of the questionnaire. Thank you for your participation!

If you have any questions, please contact Ms Anam Ahmed (researcher at Panaxea) at anam.ahmed@panaxea.eu
