# Supplementary material for: Person-centred care in the Dutch primary care setting: Refinement of middle-range theory by patients and professionals
Source: PLoS One. 2023 Mar 9;18(3):e0282802. doi: 10.1371/journal.pone.0282802 (PMC9997984; doi:10.1371/journal.pone.0282802)
Supplement: S3 File — (PDF) [file pone.0282802.s003.pdf]

Supplementary file 3. Results of Delphi round 1

Items	Median (IQR)	Consensus in 1-3 range (%)	Consensus in 4-6 range (%)	Consensus in 7-9 range (%)	Overall consensus
Context					
Setting up a personalised care planning	7 (3)	0	27	73	*Equivocal*
Preparation of consultation by patient	7 (2)	9	27	64	*Equivocal*
HCPs setting goals, making action plans, coordinating, supporting and assessing care process of patients	8 (0)	0	9	91	Relevant
Training/educating young HCPs during medical education on important aspects of person-centred care	9 (1)	0	0	100	Relevant
HCPs having the right skills (e.g., regarding communication, shared decision-making, providing culturally sensitive care)	9 (1)	0	0	100	Relevant
HCPs having the right knowledge about the epidemiology and the treatment effects in different ethnic groups	8 (0)	0	0	100	Relevant
Having better patient access to documents, recorded consultations (notes, etc.)	7 (1)	0	9	91	Relevant
Improving the accessibility of healthcare organisations	9 (1)	0	9	91	Relevant
Supporting better integration between ICT systems	8 (3)	0	36	64	*Equivocal*
Efficient use of information technology (IT)	7 (2)	0	27	73	*Equivocal*
Applying IT- and e-health initiatives	7 (2)	0	45	55	*Equivocal*
Foresee in the required capacity (time, staff, resources)	8 (2)	0	0	100	Relevant
Having sufficient male and female HCPs per practice	6 (2)	0	64	36	*Equivocal*
Offering (more) space and resources to HCPs to experiment	7 (2)	0	36	64	*Equivocal*
Patients having social support (networks)	8 (2)	0	18	82	Relevant
Having structural attention for low health skills/person-centered care in the policy of the organization	8 (2)	0	0	100	Relevant
Strengthening the quality of care through supportive health policy	8 (1)	9	9	82	Relevant
Aligning healthcare purchasing to local needs/policy	8 (1)	9	9	82	Relevant
Providing patient education	8 (1)	0	18	82	Relevant
HCPs stimulating patient empowerment	5 (4)	18	55	27	*Equivocal*
Having a good collaboration between HCPs/strong team	8 (1)	0	9	91	Relevant
Actively involving patients and patient experiences when designing care (processes)	9 (1)	0	0	100	Relevant

Involving patients in the development of new instruments (tools, step-by-step plan, booklets)	8 (1)	0	18	82	Relevant
Patients having a high/low socioeconomic status	7 (5)	27	18	55	*Equivocal*
Providing better administrative support for HCPs	7 (3)	0	45	55	*Equivocal*
HCPs having a shared vision	7 (2)	0	45	55	*Equivocal*
Using evidence-based guidelines	7 (1)	0	18	82	Relevant
Shifting the focus from a disease- and complaint-oriented approach	8 (2)	0	18	82	Relevant
Foreseeing in sufficient time for patients during consultation	9 (2)	0	18	82	Relevant
Flexible payment systems	7 (2)	0	27	73	*Equivocal*
Mechanisms					
HCPs providing effective communication	9 (1)	0	0	100	Relevant
Simplifying treatment strategies and information for patients	8 (2)	0	0	100	Relevant
Investing in understandable information material	8 (1)	0	9	91	Relevant
Encouraging patients to ask questions to HCP(s)/patients having the confidence to ask questions	8 (1)	0	9	91	Relevant
Involving family and informal caregivers in the care process	7 (1)	0	18	82	Relevant
Patients having an active role in their care process	8 (1)	0	0	100	Relevant
Stimulating patient's self-efficacy	7 (2)	0	18	82	Relevant
HCPs promoting involvement, support and reinforcement of patients	8 (2)	0	27	73	*Equivocal*
Providing self-management support	8 (1)	0	9	91	Relevant
Focus on care coordination	8 (2)	0	9	91	Relevant
Establishing a therapeutic relationship	9 (1)	0	0	100	Relevant
Achieving effective collaboration between patient and HCP(s)	8 (2)	0	9	91	Relevant
HCPs having an open and empathic attitude	8 (1)	0	9	91	Relevant
HCPs respecting the wishes and preferences of patients	8 (1)	0	0	100	Relevant
HCPs applying shared decision-making together with patients	8 (1)	0	9	91	Relevant
Have a holistic focus	8 (2)	0	18	82	Relevant
HCPs who are aware of the patient's social circumstances	9 (1)	0	9	91	Relevant
HCPs working in a culturally competent way	7 (1)	0	9	91	Relevant
Stimulating self-monitoring by patient	6 (3)	0	64	36	*Equivocal*

Outcomes					
Higher therapy adherence	8 (2)	0	0	100	Relevant
Improved patient-centred treatment/approach	9 (1)	0	9	91	Relevant
Improved intensity of support provided	8 (2)	0	36	64	*Equivocal*
Improved health-related quality of life(HRQoL)	8 (1)	0	9	91	Relevant
Improved self-management skills of patients	7 (2)	9	18	73	*Equivocal*
Higher satisfaction of patient, informal caregiver and/or HCP(s)	8 (2)	0	9	91	Relevant
Improved health outcomes	8 (1)	0	9	91	Relevant
Improved health system outcomes (reduced use of healthcare system, less referrals, less follow-up examinations, reduced emergency department visits, reduced hospital (re)admissions)	7 (2)	0	27	73	*Equivocal*
Increased patient involvement	8 (1)	0	9	91	Relevant
Higher cost-effectiveness of healthcare	8 (1)	0	18	82	Relevant
Higher quality of care	8 (2)	0	0	100	Relevant
More accessible care	8 (1)	0	18	82	Relevant
Improved relationship between patient and HCP(s)	8 (2)	0	9	91	Relevant
Improved psychological health outcomes	8 (1)	0	9	91	Relevant
