# Supplementary material for: Person-centred care in the Dutch primary care setting: Refinement of middle-range theory by patients and professionals
Source: PLoS One. 2023 Mar 9;18(3):e0282802. doi: 10.1371/journal.pone.0282802 (PMC9997984; doi:10.1371/journal.pone.0282802)
Supplement: S4 File — (PDF) [file pone.0282802.s004.pdf]

Supplementary file 4. Results of Delphi round 2

Items	Median (IQR)	Consensus in 1-3 range (%)	Consensus in 4-6 range (%)	Consensus in 7-9 range (%)	Overall consensus
Context					
Setting up a personalised care planning	7 (1)	9	36	55	*Equivocal*
Preparation of consultation by patient	6 (2)	18	64	18	*Equivocal*
Supporting better integration between ICT systems	8 (1)	9	9	82	Relevant
Efficient use of information technology (IT)	6 (3)	9	45	45	*Equivocal*
Applying IT- and e-health initiatives	7 (2)	9	36	55	*Equivocal*
Having sufficient male and female HCPs per practice	5 (2)	18	55	27	*Equivocal*
Offering (more) space and resources to HCPs to experiment	8 (1)	0	9	91	Relevant
HCPs stimulating patient empowerment	5 (4)	27	45	27	*Equivocal*
Patients having a high/low socioeconomic status	5 (5)	27	27	45	*Equivocal*
Providing better administrative support for HCPs	7 (2)	18	18	64	*Equivocal*
HCPs having a shared vision	8 (1)	9	9	82	Relevant
Flexible payment systems	8 (2)	0	9	91	Relevant
Mechanisms					
HCPs promoting involvement, support and reinforcement of patients	8 (2)	0	9	91	Relevant
Stimulating self-monitoring by patient	6 (2)	9	55	36	*Equivocal*
Outcomes					
Improved intensity of support provided	8 (1)	0	0	100	Relevant
Improved self-management skills of patients	6 (2)	9	45	45	*Equivocal*
Improved health system outcomes (reduced use of healthcare system, less referrals, less follow-up examinations, reduced emergency department visits, reduced hospital (re)admissions)	5 (1)	18	64	18	*Equivocal*
